# Enriched childhood experiences moderate age-related motor and cognitive decline

**DOI:** 10.3389/fnbeh.2013.00001

**Published:** 2013-02-18

**Authors:** Megan J. Metzler, Deborah M. Saucier, Gerlinde A. Metz

**Affiliations:** ^1^Canadian Centre for Behavioural Neuroscience, University of LethbridgeLethbridge, AB, Canada; ^2^The Dean of Science, University of Ontario Institute of TechnologyOshawa, ON, Canada

**Keywords:** bimanual motor skill, motor coordination, weather prediction task, keyboard, piano, music training, implicit learning, verbal ability

## Abstract

Aging is associated with deterioration of skilled manual movement. Specifically, aging corresponds with increased reaction time, greater movement duration, segmentation of movement, increased movement variability, and reduced ability to adapt to external forces and inhibit previously learned sequences. Moreover, it is thought that decreased lateralization of neural function in older adults may point to increased neural recruitment as a compensatory response to deterioration of key frontal and intra-hemispheric networks, particularly of callosal structures. However, factors that mediate age-related motor decline are not well understood. Here we show that music training in childhood is associated with reduced age-related decline of bimanual and unimanual motor skills in a MIDI keyboard motor learning task. Compared to older adults without music training, older adults with more than a year of music training demonstrated proficient bimanual and unimanual movement, evidenced by enhanced speed and decreased movement errors. Further, this group demonstrated significantly better implicit learning in the weather prediction task, a non-motor task. The performance of older adults with music training in those tasks was comparable to young adults. Older adults, however, displayed greater verbal ability compared to young adults irrespective of a past history of music training. Our results indicate that music training early in life may reduce age-associated decline of neural motor and cognitive networks.

## Introduction

Aging impacts many critical aspects of movement and cognition. Aging is implicated in slower reaction times (Riecker et al., 2006; Poston et al., 2009) and slower execution of movement during standardized motor tests (Ruiz et al., 2007), drawing tasks (Lee et al., 2010), and reaching movements (Rossit and Harvey, 2008; Poston et al., 2009; Verrel et al., 2012). Older adults adjust and inhibit motor movements more slowly than their younger counterparts (Potter and Grealy, 2006; Sarlegna, 2006; Rossit and Harvey, 2008) including for finger sequences (Trewartha et al., 2009) and bimanual movements (Swinnen, 1998). Older adults also demonstrate greater variability of movement compared to young adults for tapping (Bangert et al., 2010), reaching (Cooke et al., 1989), and grip force in interacting with an object (Danion et al., 2007). Evidence suggests that older adults use different strategies across a range of movements (Ketcham et al., 2004; Cesqui et al., 2008). In addition, aging is commonly associated with a decline in episodic memory, verbal ability, and non-verbal reasoning (James et al., 2011; Pillai et al., 2011). Age-related cognitive decline also involves attention and visuoperceptual abilities (Ardila et al., 2000; Yochim et al., 2009), which in turn may affect motor skill learning.

Aging is also linked to disruption of neural pathways essential to skilled movement. In diffusion tensor fMRI, older adults show axonal shrinkage, demyelination, and even axonal loss in pericallosal and callosal pathways compared to their younger counterparts (Bennett et al., 2010). Evidence suggests that these neural changes contribute to age-related movement deterioration and result in altered patterns of neural activation. Reported changes in neural activation associated with aging include increased activation of ipsilateral M1 (Naccarato et al., 2006), sensorimotor (Riecker et al., 2006) and premotor cortices (Riecker et al., 2006; Rowe et al., 2006). Older adults also exhibit more local connectivity and reduced connectivity between distant motor-related areas (Rowe et al., 2006). During bimanual movement, older adults show decreased lateralization (Przybyla et al., 2011) and additional areas of activation, such as the SMA, inferior parietal cortex, and dorsolateral prefrontal cortex, despite slower movement speed (Goble et al., 2010). Decreased lateralization of neural function in older adults may point to increased recruitment of neural resources as compensation for deterioration of frontal and key intra-hemispheric networks, particularly of callosal structures (Naccarato et al., 2006; Riecker et al., 2006; Rowe et al., 2006; Bangert et al., 2010; Bennett et al., 2010). However, factors that influence age-related motor deterioration were not clearly delineated.

The multimodal nature of music training has been shown to enhance motor skills and cognitive abilities alike (Haslinger et al., 2005; D'Ausilio et al., 2006; Forgeard et al., 2008; Moreno et al., 2011). The present study investigates the relationship between skilled motor, episodic (verbal) and implicit (weather prediction task) learning in young vs. older healthy subjects as a function of music training in childhood. Using a newly developed motor task, we show that early music training diminishes age-related decline of bi- and unimanual motor skills. Our results indicate that childhood experiences such as music training may have a protective effect on age-associated decline of neural motor networks.

## Methods

### Subjects

Twenty-seven young adults (*M* = 21.13, *SD* = 2.53) and 29 older adults (*M* = 73.03 years, *SD* = 9.59) participated in the study (Table 1). The group of young adults consisted of university-age students who participated in the study for credit in undergraduate courses through the Department of Psychology's Human Subject Pool. Older adults were recruited through a local senior center and word of mouth. Exclusion criteria included history of neurological or motor impairment and playing the piano in the past year. Four cases were excluded due to movement disorders resulting from neurological or orthopaedic conditions; one subject withdrew from the study. Ethics approval for the study was obtained from the University of Lethbridge Human Subject Committee.

**Table 1:** **Summary of demographic data of the subjects**.

	**Number of subjects**	**Age range**	**Mean age**	**Years of training (range)**	**Mean years of training**	**Piano experience**
Young M−	12 (8M, 4F)	19–28	22.58	0	0	0
Young M+	15 (6M, 9F)	18–26	20.16	1–6	2.40	2
Older M−	18 (8M, 10F)	59–95	74.67	0	0	0
Older M+	11 (5M, 6F)	55–81	70.36	1–10	3.81	7

M, male; F, female.

### Behavioral testing

**Figure 1:**
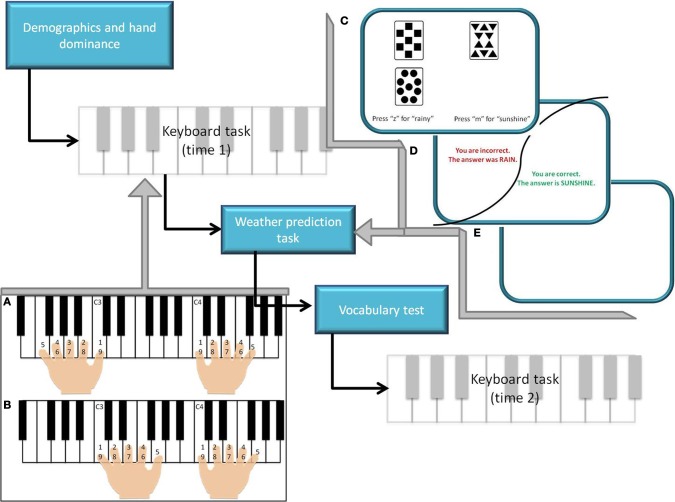
**Study procedure. (A)** Symmetrical condition of the keyboard task, with simultaneous movement of the hands in accordance with ascending numbers. **(B)** Asymmetrical condition of the keyboard task, with simultaneous movement of the fingers in accordance with ascending numbers. **(C)** Stimulus presentation for the weather prediction task (pseudorandom presentation of card combinations). **(D)** Feedback to subject response based on probabilities associated with each combination of cards. **(E)** One second inter-trial interval.

After giving informed consent, hand dominance was determined via a questionnaire (Elias et al., 1998). Both left and right handed individuals were included in the study. Participants performed several movement sequences on a MIDI keyboard, a vocabulary test (Ekstrom et al., 1976) and the weather prediction task (Figure 1). The vocabulary test asked participants to choose synonyms of target words to assess advanced vocabulary and extended range vocabulary. The weather prediction task is a probability learning task and a measure of implicit learning that involves the basal ganglia dopamine system (Knowlton et al., 1994; Rieckmann and Bäckman, 2009). Using a computer program, participants were provided with sets of different shapes and asked to predict the outcome and indicate the strategy used to solve the task.

Participants performed symmetrical and asymmetrical movements comprising of the first five notes of C major standard and contrary motion scales, respectively (Figure 1). For symmetrical and asymmetrical movements and for movements with either the dominant and non-dominant hand alone the speed [sec/keystroke] and the errors [per eight clean trials] were analysed. Participants were further divided into groups that had received a year or more of music lessons (M+) or less than a year of music lessons (M−) (26 participants, 11 older adults had received lessons; Table 1). Of those who had received music lessons in the past, older adults had on average 3.81 (1.78 SD) years of lessons, whereas younger adults had on average 2.40 (1.74 SD) years of lessons. Of the 26 participants who had taken music lessons, only 9 had taken piano lessons (Table 1). Regardless, in order to participate in the study, participants could not actively have played the piano within the past year. Further, participants had not taken music lessons since adolescence.

### Statistical analysis

Statistical analysis included Pearson's correlation coefficients and Fisher's *r* to *z*-transformation and *z*-tests (Meng et al., 1992). Furthermore, *post-hoc* analyses (Bonferroni) were performed to determine group differences. One young M+ subject was excluded from correlation analysis because the number of years of music training was not known. A *p*-value of <0.05 was chosen as the significance level.

## Results

### A history of music lessons is linked to improved manual motor performance

**Figure 2:**
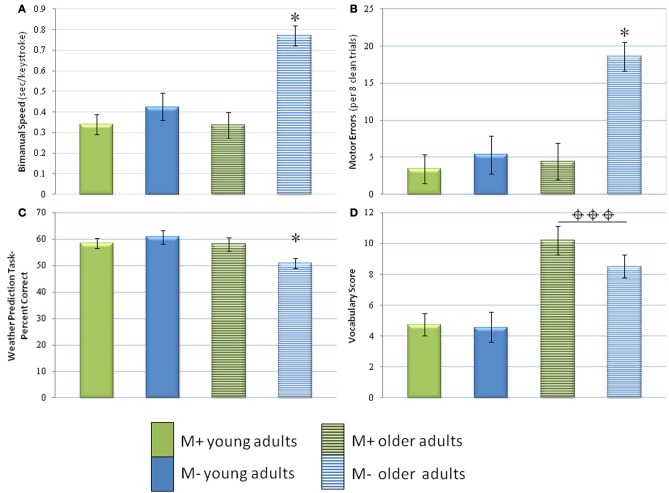
**Performance of M− and M+ older and young adults.** Results of the bimanual task (**A**, speed; **B**, errors), weather prediction task (**C**, percent correct), and vocabulary task (**D**, score) are displayed. For measures with which there was an interaction of age and history of music lessons, ^*^ denotes the group that performed significantly worse than the other groups. For measures with which there was only an effect of age, 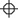

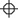

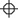
 denotes the age group that performed significantly better. Note that music training preserved bimanual performance and implicit learning in the weather prediction task. ^*^ indicates *p* < 0.05, 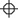

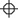

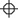
 indicates *p* < 0.001. Bars represent the means and error bars represent the standard deviation.

**Figure 3:**
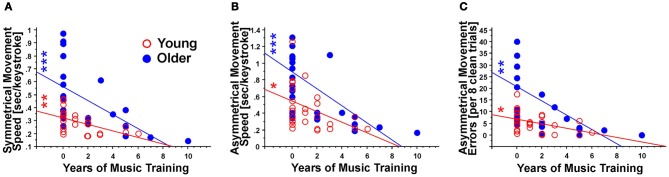
**Correlation analysis of the years of music training with motor performance in terms of (A) symmetrical movement speed; (B) asymmetrical movement speed; and (C), asymmetrical movement errors.** Note the overall negative correlation between the variables, indicating that longer music training is related to better motor performance. These correlations were stronger for older individuals than for younger ones. ^*^Indicates *p* < 0.05; ^**^Indicates *p* < 0.01; ^***^Indicates *p* < 0.001.

All analyses of bimanual and unimanual motor performance revealed an effect of past history of lessons and age group (Table 2; Figures 2A,B). Furthermore, for all variables, motor performance was negatively related to the number of years of music training in M+ subjects (Table 3; Figure 3). Thus, longer music training was associated with better movement performance in both young (*n* = 15) and older M+ subjects (*n* = 11) as outlined in the following.

**Table 2:** **Mean scores (±SDs) on the three tasks for older and young adults with and without music training (M−: no history of music lessons; M+: positive history of music lessons)**.

		**Vocabulary**	**Motor learning task**		**Weather prediction task**	
		Correct^*^	Speed^**^	Errors^**^	RT^*^	% Correct^**^
**Older adults**	M−	8.933 (3.999)	**0.770 (0.277)**	**18.654 (13.799)**	1573.386 (469.043)	**50.953 (7.055)**
	M+	10.200 (3.166)	0.337 (0.187)	4.436 (3.803)	1713.264 (416.247)	58.021 (12.261)
**Younger adults**	M−	4.580 (2.001)	0.425 (0.198)	5.333 (3.109)	1332.979 (263.386)	60.686 (7.144)
	M+	4.756 (2.403)	0.340 (0.131)	3.417 (2.404)	1308.621 (438.228)	58.388 (6.717)

Bold text indicates that the group performed significantly worse than the other 3 groups.

* Indicates a main effect of age group, p < 0.05.

** Indicates an interaction between age group and history of lessons, p < 0.05.

**Table 3:** **Summary of correlations of years of music training with manual motor performance and cognitive performance among age groups (M− and M+) and M+ participants**.

**Years of music training**	***r* Young (M− and M+)**	***r* Young (M+)**	***r* Older (M− and M+)**	***r* Older (M+)**
Symmetrical speed	−0.521^**^	−0.609^*^	−0.573^***^	−0.506
Asymmetrical speed	−0.431^*^	−0.573^*^	−0.626^***^	−0.404
Non-dominant speed	−0.387^*^	−0.541^*^	−0.640^***^	−0.656^*^
Dominant speed	−0.443^*^	−0.603^*^	−0.606^***^	−0.661^*^
Symmetrical errors	−0.396^*^	−0.484	−0.382^*^	−0.359
Asymmetrical errors	−0.409^*^	−0.419	−0.521^**^	−0.489
Non-dominant errors	−0.179	−0.125	−0.257	−0.123
Dominant errors	−0.187	−0.297	−0.434^*^	−0.268
Weather prediction %	0.120	0.333	0.077	−0.486
Weather prediction time	0.102	0.111	0.202	0.312
Vocabulary	−0.087	−0.014	0.094	−0.287

Correlation coefficients (r-values) are provided and significance levels are noted with asterisks:

* p < 0.05;

** p < 0.01;

*** p < 0.001.

In movement speed with the bimanual components of the motor learning task, the years of music training were related to faster manual performance in both symmetrical (all *p*'s > 0.01) and asymmetrical conditions (all *r*'s <−0.43, *p* > 0.05 for young, *p* > 0.0)1 for older adults; see Table 3). The correlations became stronger in young adults when only M+ subjects were included (*p* < 0.05), but weaker in older adults under the same conditions (all *r*'s <−0.57 for young, all *r*'s <−0.40 for older adults). Interestingly, *post-hoc* analyses (Bonferroni) indicated that M− older adults were significantly slower than any of the other three groups (Figure 2A). There were no significant differences among the other three groups. Exploratory analyses suggested that M− older adults performed the asymmetrical task with less speed (*M* = 0.95, *SD* = 0.35; compared to the next highest mean, M− young adults, at *M* = 0.53, *SD* = 0.31 or M+ older adults, *M* = 0.38, *SD* = 0.25).

Similarly, a past history of music lessons was associated with improved performance in a unimanual task. The speed of performance with the non-dominant and the dominant hand became faster in young and older adults with more years of music training (*r*'s < −0.39 and *p*'s > 0.05 for young; *r*'s < −0.61 and *p*'s < 0.001 for older adults; see Table 3). This association became stronger in young adults and weaker in older adults when only M+ subjects were included (all *p*'s < 0.05; Table 3). The years of music performance were strongly related to motor performance independently of the type of instrument played (see Table 4). In M+ individuals who had taken piano lessons, the associations were stronger among older adults (e.g., dominant speed *r* = −0.77, *p* < 0.05) than among all age groups combined (dominant speed *r* = −0.471, *p* < 0.05). Furthermore, performance in the unimanual task revealed that M− older adults were much slower with their dominant and non-dominant hands than any other group (all *p*'s < 0.001; dominant hand: M− older adults *M* = 0.37, *SD* = 0.11, M+ older adults *M* = 0.24, *SD* = 0.07; non-dominant hand: M− older adults *M* = 0.42, *SD* = 0.11 vs. M+ older adults *M* = 0.26, *SD* = 0.078). M− older adults moved their dominant hand significantly faster than their non-dominant hand (*p* < 0.001). The number of errors made with their dominant and non-dominant hands was also higher for M− older adults compared to any other group (all *p*'s < 0.01; dominant hand: M− older adults *M* = 4.26, *SD* = 2.87, M+ older adults *M* = 1.63, *SD* = 1.16; non-dominant hand: M− older adults *M* = 5.28, *SD* = 6.99 vs. M+ older adults *M* = 1.73, *SD* = 1.66).

**Table 4:** **Summary of correlations among the years of music training in M+ subjects concerning the past experience playing piano vs. playing other instruments**.

**Years of music training**	***r* Young and older with piano lessons**	***r* Young and older without piano lessons**
Symmetrical speed	−0.382	−0.459^*^
Asymmetrical speed	−0.405	−0.454^*^
Non-dominant speed	−0.473^*^	−0.480^*^
Dominant speed	−0.471^*^	−0.479^*^
Symmetrical errors	−0.271	−0.248
Asymmetrical errors	−0.281	−0.429
Non-dominant errors	−0.044	−0.114
Dominant errors	−0.279	−0.160

Correlation coefficients (r-values) are provided and significance levels are noted with asterisks:

* p < 0.05.

The analysis of movement errors revealed again a negative association between the past history of music lessons and performance in both bimanual and unimanual conditions (Tables 2 and 3). This association became significant for the age effect when including both M− and M+ subjects (*r*'s < 0.38, *p*'s < 0.05). Thus, among all participants music experience in childhood was associated with lower error rates in the symmetrical and asymmetrical conditions of the bimanual task. Furthermore, older adults with music training displayed reduced error rates in the dominant hand (*r* = −0.43, *p* < 0.05; Table 3). *Post-hoc* analyses (Bonferroni) indicated that M− older adults made significantly more errors than any of the other three groups (*p* < 0.05; Figure 2B). There were no other significant differences among the other three groups.

### Music training is associated with enhanced implicit learning ability

For the weather prediction task, correlational analysis revealed an overall positive relationship between past history of lessons and the percentage of correct responses (all *r*'s > 0.09). *Z*-scores revealed no significance. Notably, *post-hoc* analyses (Bonferroni) indicated that M− older adults performed significantly more poorly than the other three groups (all *p*'s < 0.05; Figure 2C). No other significant differences were found.

In terms of the time needed to complete the weather prediction task, correlations revealed a positive, but no significant relationship. Older adults took more time to complete the trials correctly than young adults (*p* < 0.01; older adults, *M* = 1626.44, *SD* = 447.46; younger adults, *M* = 1317.32, *SD* = 379.84; Table 2).

Two-Tailed tests revealed a significant negative relationship between the percent correct in the weather prediction task and symmetrical movement speed (*p* < 0.01), asymmetrical movement speed (*p* < 0.01), and asymmetrical error (*p* < 0.05). Furthermore, the reaction time in the weather prediction task was positively correlated with asymmetrical movement speed (*p* < 0.001), symmetrical movement error (*p* < 0.05), and asymmetrical movement error (*p* < 0.01).

In the vocabulary task, performance was positively related to the past history of music lessons (*r* = 0.08) in older adults, and negatively in younger adults, although the correlation was not significant. Performance in the vocabulary task was also positively related to reaction time in the weather prediction task among all ages and groups combined (*p* < 0.05, *r* = 0.26), but not to motor performance. Importantly, older adults (*M* = 9.17, *SD* = 3.74) performed significantly better than young adults in the vocabulary task (*p* < 0.001; *M* = 4.69, *SD* = 2.23; Table 2, Figure 2D).

## Discussion

The present data show that in the absence of differences in bimanual performance among M− and M+ young adults, M+ older adults performed significantly better than M− older adults in the keyboard task. This change occurred regardless of the instrument learned and was not influenced by previous experience playing the piano. The absence of a significant difference in performance between M+ older adults and young adults suggests that the protective effect of beneficial childhood experiences, including music training, is durable and potent. Remarkably, M− older adults also performed significantly more poorly than other groups on a measure of implicit learning, the weather prediction task, in terms of percentage of correct responses. Consequently, these differences cannot solely be attributed to preservation of peripheral motor systems as a result of music training, but must involve central neural systems. Nevertheless, the results of the vocabulary test do not point to innate differences between M− and M+ older adults, and confirm aging-related improvement in semantic knowledge (Verhaeghen, 2003). Consequently, our research suggests that music training selectively alters neural systems implicated in motor learning in the absence of global neurological change.

The present results are in line with studies suggesting a link between motor and cognitive processing in older adults. For example, older adults exhibit temporal declines of bimanual circle drawing and simultaneous tapping (Bangert et al., 2010). Interestingly, lower executive function correlated with asynchronous inter-manual timing deficits, and better performance of the most difficult bimanual circling task was associated with better working memory for older adults (Bangert et al., 2010). This led to the postulation that for the older adults, executive and working memory functions are engaged for difficult tasks (Bangert et al., 2010).

The weather prediction task may provide a means of investigating the integrity of neural structures necessary for manual movement. Further, the results also suggest that music training in childhood may reduce the global decline of central dopamine function normally associated with aging (Rieckmann and Bäckman, 2009). While the weather prediction task may reflect the integrity of the basal ganglia dopamine system (Moody et al., 2004), recent studies have challenged the notion of impaired performance associated with deterioration of the neostriatum (Wilkinson et al., 2008; Jahanshahi et al., 2010). Alternatively, it may be argued that performance of a bimanual keyboard task and the weather prediction task rely on shared patterns of connectivity that are strengthened by music training. The rather weak correlation between performance in these tasks argues for the involvement of both shared and unshared neuronal substrates.

While the present data suggest a positive influence of early music training on age-related functional decline, other childhood experiences may contribute to these outcomes. An environment enriched with music lessons may be associated with other exposures that support behavioral, physiological, and neurological development, such as tactile stimulation (Sharp et al., 2012), nutritional or social variables (Forns et al., 2012). Furthermore, lifetime leisure activities (James et al., 2011; Pillai et al., 2011; Gow et al., 2012), stress (Segovia et al., 2009; Merrett et al., 2010), physical activity (Buchman et al., 2012), social activity (Buchman et al., 2009), and education (Ardila et al., 2000; Van Gerven et al., 2012) may individually or synergistically affect the rate of age-related functional decline. Accordingly, animal studies have shown beneficial effects of environmental enrichment on skilled motor function (Jadavji et al., 2006; Jadavji and Metz, 2009; Knieling et al., 2009), age-related memory decline and neuronal plasticity (Leal-Galicia et al., 2008; Segovia et al., 2009). Notably, enrichment results in increased myelination of aged rat corpus callosum (Zhao et al., 2012), a structure central to bimanual coordination and music performance (Schlaug et al., 1995).

Prior research points to a neural mechanism which may underpin our findings. It is established that music training results in activation of an extensive network that includes auditory and sensorimotor networks (Bangert and Altenmüller, 2003; Haslinger et al., 2005; Bangert et al., 2006; D'Ausilio et al., 2006). Whether music training prevents age-related deterioration of the underlying anatomical structures and pathways or simply provides an effective means of compensation remains unclear at this point. Ultimately, music training results in macrostructural changes to the brain, specifically of the primary motor and somatosensory cortices, inferior temporal gyri, anterior corpus callosum, and cerebellar cortex (Elbert et al., 1995; Schlaug et al., 1995; Gaser and Schlaug, 2003; Gentner et al., 2010). Hence, music training results in differential augmentation of key areas of the motor network, including callosal structures. Accordingly, symmetrical movement is shown to rely on efficient input from the dominant to the non-dominant hemisphere for movement (Maki et al., 2008; Walsh et al., 2008). Presumably, strengthening of neural connections between these key areas likely plays a vital role in preservation of manual motor learning and performance in the long term. The present results suggest that music training early in life may attenuate the decline of neural motor and cognitive networks associated with advanced age.

## Author contributions

Megan J. Metzler, Deborah M. Saucier, and Gerlinde A. Metz designed the study. Megan J. Metzler wrote computer code for data extraction and collection, recruited subjects and collected the data. Megan J. Metzler and Gerlinde A. Metz wrote the manuscript and created figures. Deborah M. Saucier and Gerlinde A. Metz completed the statistical analysis, wrote the results, and provided equipment and software.

### Conflict of interest statement

The authors declare that the research was conducted in the absence of any commercial or financial relationships that could be construed as a potential conflict of interest.
